# The Leucine Zipper Domains of the Transcription Factors GCN4 and c-Jun Have Ribonuclease Activity

**DOI:** 10.1371/journal.pone.0010765

**Published:** 2010-05-21

**Authors:** Yaroslav Nikolaev, Christine Deillon, Stefan R. K. Hoffmann, Laurent Bigler, Sebastian Friess, Renato Zenobi, Konstantin Pervushin, Peter Hunziker, Bernd Gutte

**Affiliations:** 1 Biochemisches Institut der Universität Zürich, Zürich, Switzerland; 2 Biozentrum der Universität Basel, Basel, Switzerland; 3 Organisch-Chemisches Institut der Universität Zürich, Zürich, Switzerland; 4 Department of Chemistry and Applied Biosciences, Swiss Federal Institute of Technology, Zürich, Switzerland; 5 Functional Genomics Center Zürich, Zürich, Switzerland; Victor Chang Cardiac Research Institute (VCCRI), Australia

## Abstract

Basic-region leucine zipper (bZIP) proteins are one of the largest transcription factor families that regulate a wide range of cellular functions. Owing to the stability of their coiled coil structure leucine zipper (LZ) domains of bZIP factors are widely employed as dimerization motifs in protein engineering studies. In the course of one such study, the X-ray structure of the *retro*-version of the LZ moiety of yeast transcriptional activator GCN4 suggested that this *retro*-LZ may have ribonuclease activity. Here we show that not only the *retro*-LZ but also the authentic LZ of GCN4 has weak but distinct ribonuclease activity. The observed cleavage of RNA is unspecific, it is not suppressed by the ribonuclease A inhibitor RNasin and involves the breakage of 3′,5′-phosphodiester bonds with formation of 2′,3′-cyclic phosphates as the final products as demonstrated by HPLC/electrospray ionization mass spectrometry. Several mutants of the GCN4 leucine zipper are catalytically inactive, providing important negative controls and unequivocally associating the enzymatic activity with the peptide under study. The leucine zipper moiety of the human factor c-Jun as well as the entire c-Jun protein are also shown to catalyze degradation of RNA. The presented data, which was obtained in the test-tube experiments, adds GCN4 and c-Jun to the pool of proteins with multiple functions (also known as moonlighting proteins). If expressed *in vivo*, the endoribonuclease activity of these bZIP-containing factors may represent a direct coupling between transcription activation and controlled RNA turnover. As an additional result of this work, the *retro*-leucine zipper of GCN4 can be added to the list of functional *retro*-peptides.

## Introduction

Leucine zippers [1] are parallel alpha-helical coiled coil motifs and as such one of the most common mediators of protein-protein interactions [2]. The most widely known leucine zipper (LZ) proteins are the basic-region leucine zippers (bZIP) [1], which account for more than 51 unique members in *Homo sapiens* [3], comprising the second-largest family of dimerizing transcription factors in humans after bHLH proteins [4].

**Figure 1:**
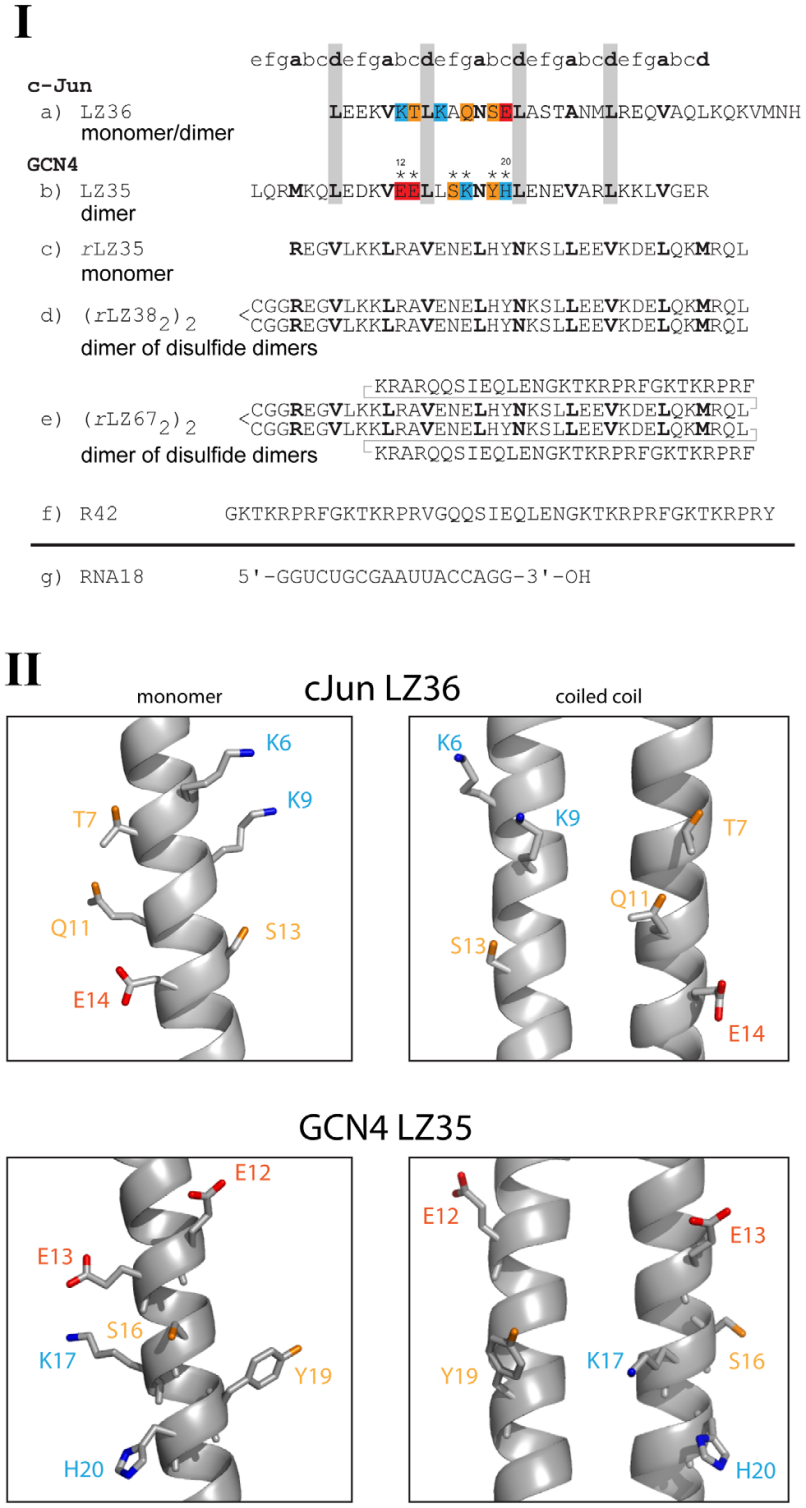
Synthetic peptides and RNA employed in the study. (**I**) Sequences of leucine zippers and RNA18. The a- and d-positions of the repeating heptads of the GCN4 and c-Jun leucine zippers are shown in bold print. In the *retro*-sequences, a- and d-positions are reversed. In the wild-type sequences (a, b) leucine residues in d-positions are highlighted grey; anionic, cationic and polar residues in the proposed active sites are highlighted red, blue and orange, respectively. a) c-Jun LZ36 (residues 280-315); b) GCN4 LZ35 (residues 247–281), asterisk (*) indicates positions of point mutations; c) GCN4 *r*LZ35, *retro*-sequence of LZ35; d) GCN4 *r*LZ38, obtained through N-terminal extension of *r*LZ35 by CysGlyGly; e) *r*LZ67, obtained through fusion of *r*LZ38 with shortened HIV enhancer-binding peptide R42; f) R42, artificial HIV enhancer-binding peptide; g) RNA18. (**II**) Topological arrangement of side chains in the proposed catalytic sites of c-Jun LZ36 (PDB: 1JUN) and GCN4 LZ35 (PDB: 2ZTA). Both monomer and coiled coil active site arrangements are shown. Residue color code corresponds to that of c-Jun and GCN4 sequences in (**I**).

Simplicity of the coiled coil fold together with high stability of the LZ motifs facilitated their extensive adoption in protein engineering studies [5], [6], [7], [8]. In one such study, a GCN4 (yeast transcription activator) *retro*-leucine zipper [Fig. 1(I c)] seemed to be most suitable as dimerization module for an artificial HIV (Human Immunodeficiency Virus) enhancer-binding peptide. To ensure dimerization of this *retro*-leucine zipper it was extended at the N-terminus by the tripeptide sequence Cys-Gly-Gly [Fig. 1(I d)] which allowed formation of a disulfide bond. Molecular weight studies in the ultracentrifuge and the crystal structure revealed that the disulfide peptide formed a noncovalent dimer or four-helix bundle and that neighbouring bundles were bridged by histidine side chains (Fig. 2C in [9]). The observed juxtaposition of histidines was vaguely reminiscent of the active-site structure of ribonuclease A (RNase A) [10] and prompted us to test the *retro*-leucine zipper for ribonuclease activity. Surprisingly, weak ribonuclease activity distinct from that of RNase A was not only found for the GCN4 *retro*-leucine zipper but also for the authentic leucine zipper domains of GCN4 and oncoprotein c-Jun (component of transcription factor AP-1).

**Figure 2:**
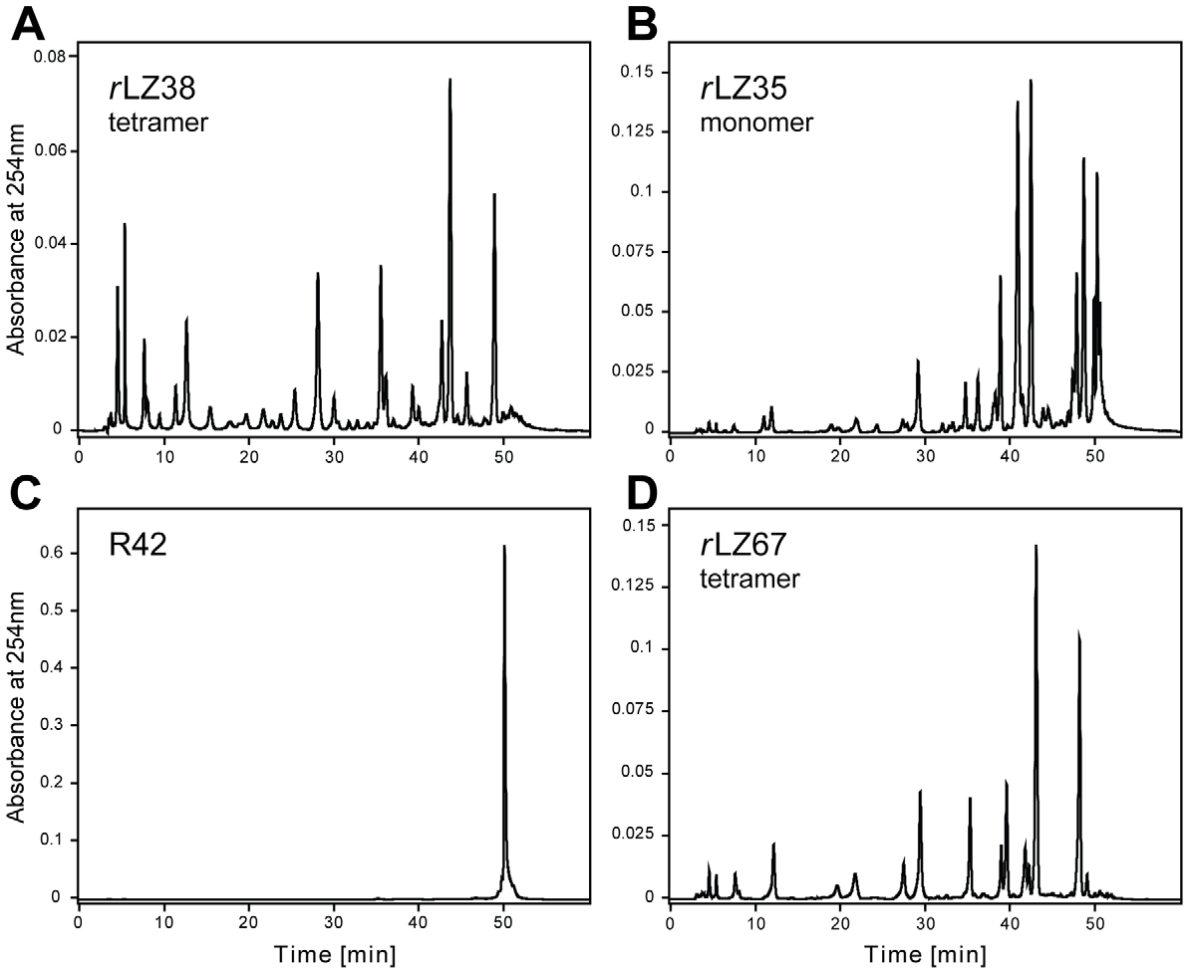
HPLC fractionation of RNA degradation products. (**A**) Cleavage products of 85 µ*M* RNA18 formed within 24 hours in presence of 28 µ*M* GCN4 *r*LZ38, (**B**) 56 µ*M* GCN4 *r*LZ35, and (**D**) 28 µ*M r*LZ67. (**C**) The mixture of R42 (14 µ*M*) and RNA18 (48 µ*M*) served as negative control. Reactions were performed in 20 m*M* Tris-HCl/80 m*M* KCl at pH 7.2 and 37°C. Retention time of uncleaved RNA18 was 50 min.

There are natural polypeptides and proteins reported to possess “moonlighting” endoribonuclease activity [11]. Examples are the homodimer of a 30-residue single zinc finger motif of the human male-associated ZFY transcription factor [12], the 278-residue zinc-alpha 2-glycoprotein [13] and conserved, approximately 100-residue DYW domains [14] of land plant pentatricopeptide repeat (PPR) proteins [15], [16]. The ZFY zinc finger cleaves single-stranded RNA with *k*_cat_ = 0.025 per min, comparable to the catalytic efficiency of group II intron ribozymes (*k*_cat_ = 0.03 per min) [17]. The zinc-alpha 2-glycoprotein also cleaves single-stranded RNA. The DYW domains of the PPR proteins have been implicated in catalyzing RNA editing in plant organelles; in addition, several recombinant DYW domains were found to possess weak endoribonuclease activity. It remains to be seen whether the ribonuclease activity of the ZFY zinc finger and the PPR DYW domains is preserved in their parent proteins and, eventually, is of biological importance.

RNA is also cleaved by diverse artificial catalysts, for example imidazole and guanidinium compounds, transition metal complexes, and designed peptides. Both the GCN4 and c-Jun leucine zipper peptides contain histidine and arginine residues. These residues are essential for the RNase A-catalyzed digestion of RNA and for specific protein-RNA binding, respectively. Therefore the presence of catalytically active imidazole- and guanidinium-containing compounds in the ribonuclease assays of the leucine zipper peptides had to be avoided. It had been shown that imidazole is active as 2-aminobenzimidazole (with a guanidinium group in disguise) [18] or when bound to beta-cyclodextrin [19], polyamines [20] or antisense oligonucleotides [21], [22]. The guanidinium group has ribonuclease activity as a bridged dimer [23]. However, the presence in our experiments of these imidazole and guanidinium derivatives and of biogenic amines (spermine, spermidine) [24] which also have weak ribonuclease activity could be excluded. This applied also to active complexes of transition metals such as Cu, Zn, and lanthanides [22], [25] because leucine zipper sequences do not contain metal-chelating motifs.

Designed peptides with RNA-hydrolyzing activity include Ni-chelating tripeptides [26], metal-free tri- and tetrapeptides containing Arg, His, Lys and Glu [27], and helical polypeptides containing basic and hydrophobic amino acid residues (Lys and Leu; [28]). However, peptides of this kind could not form during the synthesis of the leucine zipper sequences.

Catalytic hydrolysis of RNA is an essential part of RNA turnover and decay and thus a major mechanism for gene expression control [29], [30]. If the bZIP leucine zipper portions of transcription factors GCN4 and c-Jun were active *in vivo*, they may contribute to the ribonuclease activities controlling gene expression and RNA biogenesis in the nucleus, the major site of RNA turnover [31], [32], [33]. Transcription factors containing leucine zipper or zinc finger motifs would thus combine transcription activation with slow RNA degradation.

In this work we have focused on the ribonuclease activity of the authentic leucine zippers of the bZIP regions of yeast GCN4 and human c-Jun. We show that the activity of these leucine zippers is not affected by a specific inhibitor of the RNase A family but by mutations in the amino acid sequence, thus attributing the observed RNA cleavage to the synthetic peptides. In addition, our experimental data show that this activity is preserved in full-length c-Jun.

## Results

Highest priority was paid to the purity of all materials used in the ribonuclease activity assays to exclude contamination by ribonuclease-active compounds at all experimental stages. The RNase activities reported were observed for several different syntheses of the peptides, prepared in our laboratory and by external suppliers. The water used for the ribonuclease assay buffers and LC column eluents was ribonuclease-free (commercial RNase/DNase-free water or ultrafiltered water assayed for the lack of RNA hydrolytic activity). The peptides had been repeatedly exposed to basic (20% piperidine) and acidic (95% trifluoroacetic acid) conditions during solid phase synthesis and work-up and were shown by amino acid analysis, HPLC, and mass spectrometry to be homogeneous. Cleavage of the RNA substrates by the leucine zipper peptides was assayed by monitoring the time-dependent depletion of the substrates using HPLC. Prior to each activity assay a substrate control was run through the HPLC column; even after 96-h incubations in the same reaction buffer only a single sharp peak eluted at the position of the intact RNA substrate. This indicated that substrate, buffers and HPLC system were not contaminated by ribonucleases. Handling of the enzymatically active and inactive peptides during synthesis and purification was identical. In control experiments, R42 (a synthetic HIV-1 enhancer-binding peptide similar in size to the leucine zippers but strongly cationic), bovine serum albumin (BSA), glyceraldehyde-3-phosphate dehydrogenase and glucagon were inactive in 2-h to 48-h incubations with RNA substrate. Furthermore, the ribonuclease activity of the GCN4 and c-Jun leucine zippers was not affected by the ribonuclease A family-specific inhibitor RNasin. Finally, the strongest evidence for the intrinsic ribonuclease activity of the GCN4 leucine zipper peptide comes from the fact that several of its alanine mutants were inactive albeit their synthesis, purification and assaying procedures were identical to those of the wild-type peptide.

### Assays of the ribonuclease activity of wild-type and mutant GCN4 leucine zipper peptides

The first assays were performed with GCN4 *r*LZ38, the parallel four-helix bundle [9] with possible ribonuclease activity, and a commercial octadecaribonucleotide (RNA18) [Fig. 1 (I g)] as substrate. Based on the positive result of these assays [Fig. 2 (A)], single-chain *r*LZ35 ([Fig. 1(I c)], net charge +2) and tetrameric *r*LZ67 ([Fig. 1(I e)], net charge +11 per chain), the fusion peptide of *r*LZ38 with a shortened version of R42, a designed HIV enhancer-binding peptide, were tested for ribonuclease activity and were also found to cleave RNA18 [Fig. 2(B, D)]. R42 itself [Fig. 1(I f)] [34], [35], [36], a 42-residue strongly HIV enhancer-binding peptide (net charge +14), was inactive [Fig. 2(C)]. This allowed to attribute the ribonuclease activity of *r*LZ67 to the *retro*-leucine zipper component of the fusion peptide and it showed that the activity of the GCN4 *retro*-leucine zipper was preserved in a larger, heterologous sequence context. The sequence of *r*LZ67 [Fig. 1(I e)] comprised *r*LZ38 [Fig. 1(I d)] followed by the positively charged flexible linker KRAR and the C-terminal 25 residues of R42 [Fig. 1(I f)].

**Figure 3:**
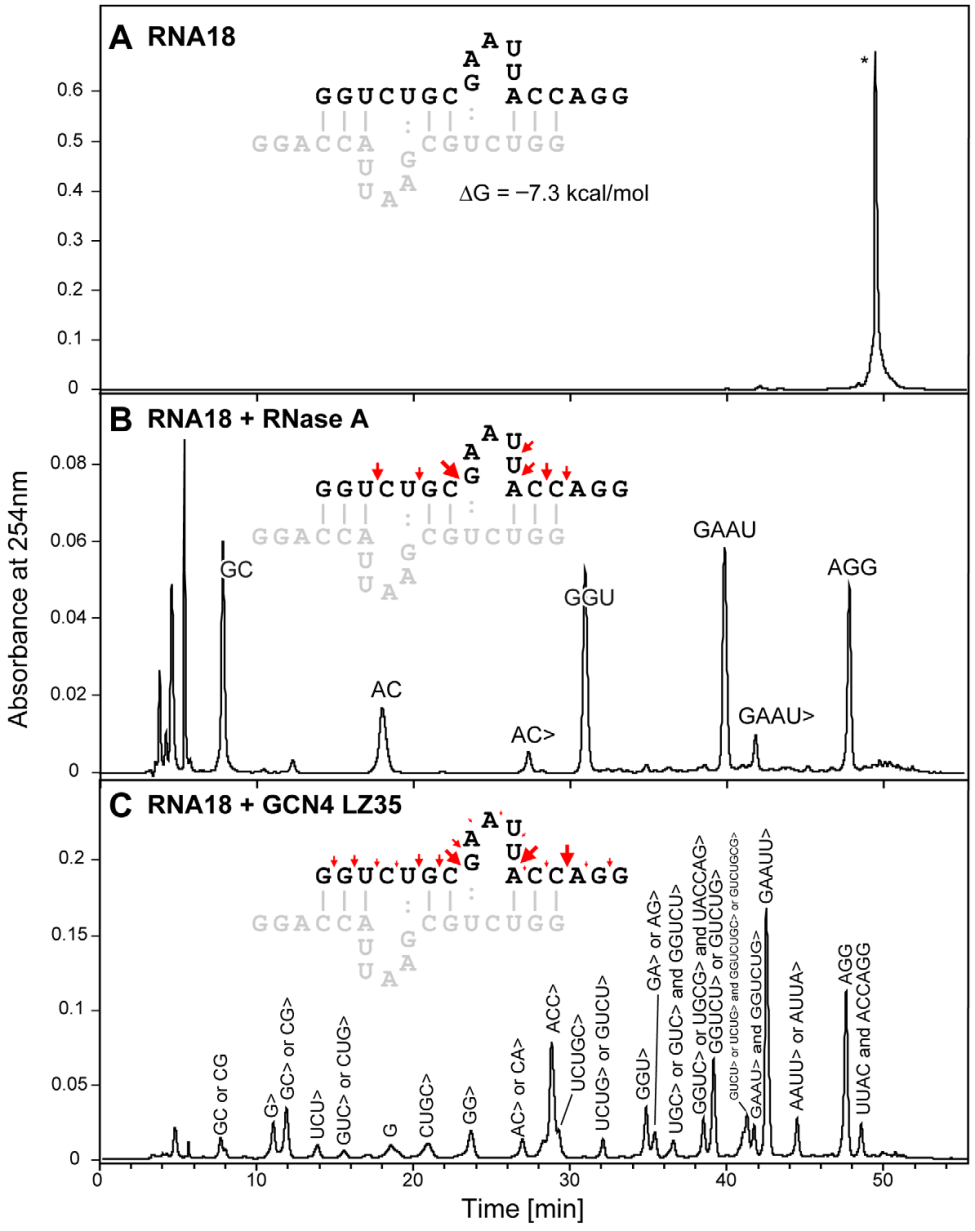
Comparison of RNA18 cleavage by GCN4 LZ35 and RNase A. RNA18 concentration was 80 µ*M*. Samples were incubated in 20 m*M* Tris-HCl/80 m*M* KCl, pH 7.2, at 37°C. The cleavage products obtained were separated by HPLC and characterized by electrospray ionization mass spectrometry. Retention time for uncleaved RNA18 was approximately 50 min. (**A**) RNA18 blank (incubation time, 24 h). (**B**) RNA18 + RNase A (90 n*M*; incubation time, 3 h); the major products were AGG (956 Da) lacking phosphate at the 3′-end, and the 3′-phosphate-containing GAAU (1′326 Da), GGU (1′013 Da), AC (651 Da), and GC (667 Da). (**C**) RNA18 + GCN4 LZ35 (50 µ*M*; incubation time, 24 h); the major products were AGG (956 Da) lacking phosphate at the 3′-end, GAAUU> (1′614 Da), GUCUG> or GGUCU> (1′606 Da), and ACC> (938 Da); the composition of several of the minor products indicated cleavage of RNA18 at the 3′-end of guanosine phosphate or adenosine phosphate residues, for example: AAUU> or AUUA>, UACCAG>, GA> or AG>, UCUG> or GUCU>, GG>, G, UCU>, and G> (“>” represents 2′,3′-cyclic phosphate group). The largely dimeric structure of RNA18 (ΔG = −7.3 kcal/mol) was established using MFOLD software [37]. Cleavage efficiencies at particular RNA bonds are shown as arrows above the RNA18 sequence. Size of arrows reflects the concentration of oligonucleotides with the corresponding 3′- and 5′-termini relative to the combined concentration of all reaction products. Product concentrations are calculated as absorbance peak integrals normalized by extinction coefficients of the corresponding oligonucleotides.

The digestion patterns produced by the *retro*-leucine zippers [Fig. 2(A, D)] and the authentic leucine zipper of GCN4 [Fig. 3(C)] in 24-h reactions using RNA18 as substrate were remarkably similar. Results of the cleavage of RNA18 by wild-type and mutant 35-residue leucine zipper peptides are summarized in Table 1. Full-length RNA18 is predicted to form a double-stranded structure with 50% of its sequence being involved in the base-pairing interactions (see below and [Fig. 3 inset]). To avoid uncertainties about the proportions of oligomeric states of the leucine zipper peptides and RNA in the degradation experiments of this study, the concentrations given for the peptides and RNA are uniformly those of the monomeric species. The cleavage percentages reflect the decrease in the integral area of the full-length 18-mer RNA substrate peak. These percentages do not take into account the subsequent secondary cleavages of smaller RNA fragments during the reaction and are therefore underestimations of the actual number of cleavages of individual phosphodiester bonds.

**Table 1:** RNA18 cleavage efficiency by Wild-Type (wt) and Mutant GCN4 LZ35^a^.

LZ peptide	% cleaved (2 h)	% cleaved (13 h)
wt	17	47
E12A	0	2
E12Q	2	6
E13A	n.d.^b^	12
S16A	<1	9
S16T	<1	11
K17A	4	47
Y19A	13	40
H20A^c^		

^*a*^Percent RNA cleaved after 2 h and 13 h, respectively, based on the amount present at the beginning of the experiment (50 µ*M*) and determined by HPLC at 254 nm. The concentration of the GCN4 leucine zippers was 30 µ*M*. Cleavages were performed in 20 m*M* Tris-HCl/80 m*M* KCl, pH 7.2, at 37°C.

^*b*^Not determined.

^*c*^Assay commented in Results.

In 20 m*M* Tris-HCl and 80 m*M* KCl, pH 7.2, wild-type GCN4 LZ35 [Fig. 1(I b)] (30 µ*M*) cleaved 47% of RNA18 (50 µ*M*) in 13 h at 37°C. Under the same conditions, the GCN4 LZ peptide with Glu12 replaced by alanine was completely inactive. Low activity (6 to 12% cleavage in 13 h) was retained by the Glu12Gln, Glu13Ala, Ser16Ala, and Ser16Thr analogues whereas exchange of Lys17 and Tyr19 for alanine did not affect the enzymatic activity (47% and 40%, respectively). In the presence of the His20Ala mutant the area of the RNA18 HPLC peak was unchanged after a reaction time of 2 h but had decreased by approximately 40% after 13 h without formation of detectable cleavage products. This indicated that the His20Ala mutant had very little if any activity and pointed to time-dependent aggregation of His20Ala - RNA18 complex(es). The latter could be reversed by dilution in 80 m*M* phosphate, carbonate, or Tris-HCl and heating of the assay mixture to 65°C before application to the HPLC column [supplementary material, Fig. S4(E,F)].

The activity maximum of the GCN4 leucine zipper peptides was at approximately pH 7 and at KCl concentrations between 75 and 100 m*M*. The presence of 1 m*M* EDTA did not affect the results of the activity assays.

The products of a 24-h digestion of RNA18 catalyzed by GCN4 LZ35 were separated and characterized using HPLC-electrospray ionization mass spectrometry and were compared with those of a 3-h digestion by RNase A [Fig. 3(B,C)]. LZ35 cleaved the RNA18 substrate mainly at the 3′-end of U and C and to a smaller extent at the 3′-end of A and G. Almost all products were obtained as the 2′,3′-cyclic phosphates [Fig. 3(C)]. In contrast, RNase A could not cleave RNA at the 3′-end of G and catalyzed the hydrolysis of the intermediate 2′,3′-cyclic phosphates to give the final 3′-phosphate-containing products [Fig. 3(B)]. Even after 96 h no degradation of RNA18 substrate was detected in the absence of LZ35 or RNase A [Fig. 3(A)].

Based on the sequence composition, RNA18 is predicted to form a stable double-stranded structure with 50% of nucleotides being involved in the base-pairing interactions ([Fig. 3(A), inset]; ΔG = −7.3 kcal/mol) [37]. The corresponding *K*_d_ of ∼7 µM at 37°C establishes that RNA18 is largely double-stranded under the initial conditions of the ribonuclease assays. Analysis of RNA18 degradation products suggests that GCN4 LZ35 avoids duplex structures, preferably targeting the flexible single-stranded regions of RNA (red arrows in [Fig. 3(C), inset]).

The ribonuclease activity of GCN4 LZ35 was confirmed using commercial RNA (baker's yeast; Sigma) as substrate [Fig. S5]. In this experiment the concentrations of RNA, leucine zipper peptide, and the BSA control were 22 µ*M* (0.5 mg/mL), 30 µ*M* (0.13 mg/mL), and 7.35 µ*M* (0.5 mg/mL), respectively, in 50 m*M* sodium acetate, pH 5; the reaction time was 24 h and the temperature 25°C. The results of this experiment were unequivocal: GCN4 LZ35 cleaved baker's yeast RNA whereas BSA was inactive.

### Ribonuclease assays of GCN4 LZ35 in presence of RNase A inhibitor

Five units of the recombinant RNase A inhibitor RNasin (Promega) did not affect the RNA18-cleaving activity of 12.5 µL of a 50 µ*M* solution of GCN4 LZ35 whereas 150 units of the inhibitor lowered the activity by approximately 60%. The RNA18 concentration at the start of the experiments was 50 µ*M*, incubation time was 13 h. In a control experiment, five units of RNasin abolished the activity of 12.5 µL of a 9 p*M* solution of RNase A completely. The effect of the inhibitor on RNA18 cleavage by GCN4 LZ35, RNase A, and RNase T_1_ is illustrated in electronic supplementary material, Fig. S3.

### Ribonuclease assays of the leucine zipper of c-Jun [Fig. 1(I a)] and full-length c-Jun

**Figure 4:**
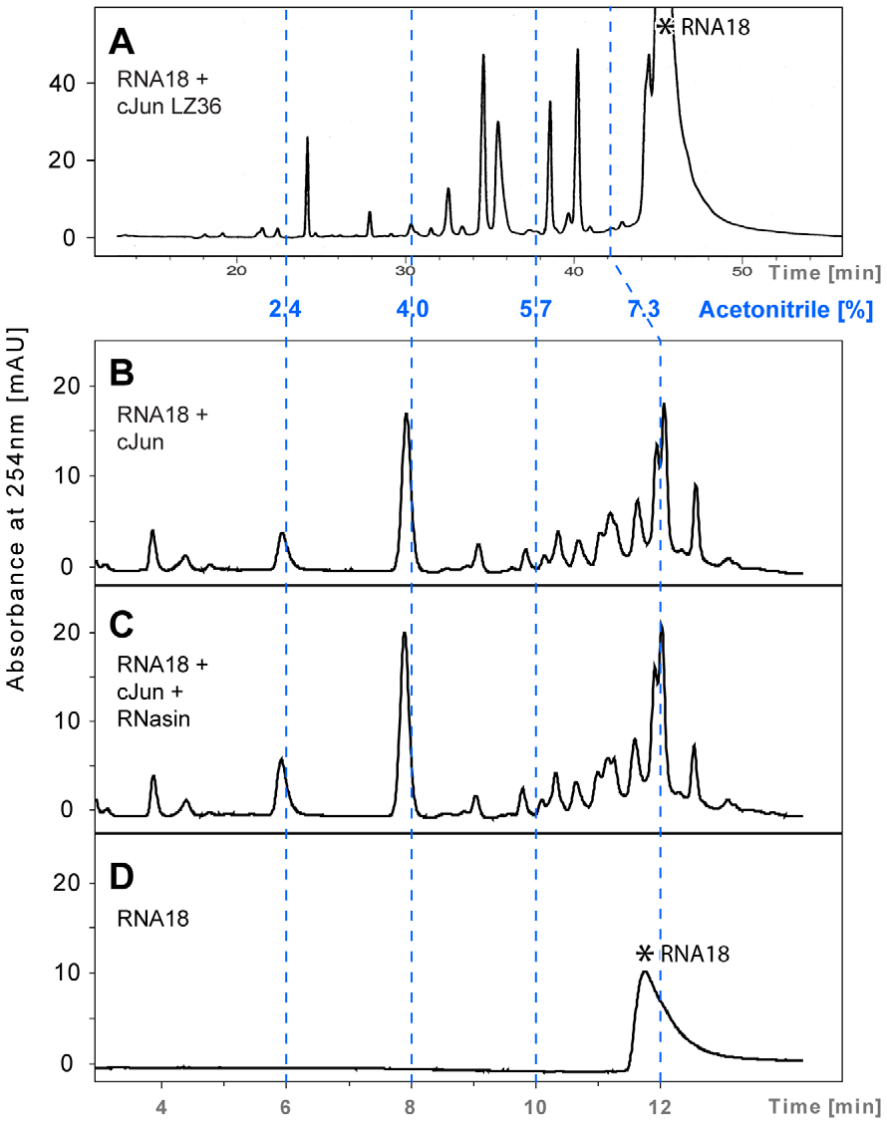
Comparison of chromatograms illustrating RNA18 cleavage by synthetic c-Jun LZ36 and full-length 40 kDa recombinant c-Jun. Reactions were performed in 20 m*M* Tris-HCl/80 m*M* KCl, pH 7.2, at 37°C. (A) c-Jun LZ36 (60 µ*M*) + RNA18 (170 µ*M*); reaction time, 4 h; column, 3.14 mL Nucleosil C-18 300-5 (Macherey & Nagel). (B) Recombinant c-Jun (20 µ*M*) + RNA18 (90 µ*M*); reaction time, 48 h; column, 2.5 mL Eclipse XDB C-18 (Agilent). (C) c-Jun + RNA18 (as in B) in the presence of 1 U of the ribonuclease A inhibitor RNasin. (D) RNA18; incubation time, 48 h. Resolution of the digestion products by the two different HPLC columns required a different gradient set-up (30-mL gradient in panel A versus 13-mL gradient in panels B, C and D). The elution profiles are presented relative to the percentage of acetonitrile in the two gradients (dashed blue lines). Asterisk (*) marks the position of intact RNA18.

Fig. 4 shows the HPLC chromatograms of the cleavage products of RNA18 produced by synthetic 36-residue leucine zipper of c-Jun (LZ36) and full-length recombinant c-Jun. In 20 m*M* Tris-HCl/80 m*M* KCl, pH 7.2, at 37°C, 60 µ*M* synthetic c-Jun LZ36 degraded 28% of 170 µ*M* RNA18 in 4 h [Fig. 4(A)] and 63% in 24 h. Under the same conditions, 20 µ*M* full-length recombinant c-Jun (Promega) cleaved approximately 50% of 90 µ*M* substrate in 48 h [Fig. 4(B,C,D)]. The prolonged reaction time chosen for the latter assay was to compensate for the lower concentration of recombinant c-Jun in these experiments (20 µ*M* c-Jun versus 60 µ*M* c-Jun LZ36). The cleavage patterns obtained showed similarities but were not identical [Fig. 4(A and B)]. The experiments with recombinant c-Jun were preliminary and were merely performed to see if the ribonuclease activity of the c-Jun leucine zipper was preserved in the full-length protein. Importantly, under the same conditions used in the inhibition experiments with GCN4 LZ35 (i.e., five units of RNasin in 12.5 µL of digestion mixture) the ribonuclease activity of recombinant c-Jun and synthetic c-Jun LZ36 was not affected. Activity assays of c-Jun LZ36 in presence of 150 units of the inhibitor were not performed.

### Kinetic analysis of RNA18 cleavage by the LZ peptides

Degradation rates of full-length 18-mer RNA substrate were used to determine the lower boundaries for the second-order catalytic rate constants (*k*_2_ > *k*_2obs_ = *V*_obs_/([LZ]•[RNA]), *M* ^−1^ min^−1^) and turnover numbers (*k*_cat_ > *k*_obs_ = *V*_obs_/[LZ], min^−1^) of phosphodiester bond cleavage by LZ peptides. Based on the ribonuclease activity assays, the minimal turnover numbers for GCN4 LZ35, c-Jun LZ36 and full-length c-Jun were 0.0024, 0.0033 and 0.0008 min^−1^, respectively (Table 2).

**Table 2:** Minimal turnover numbers and second-order rate constants of the LZ-catalyzed RNA18 degradation^a^.

	peptide	RNA18	cleavage	time	*V*_obs_	*k*_obs_ (≤*k*_cat_)	*k*_2obs_ (≤*k*_2_)
	µ*M*	µ*M*	%	h	n*M* min^−1^	min^−1^	*M* ^−1^ min^−1^
GCN4 LZ35	30	50	17	2	70.8	0.0024	47
cJun LZ36	60	170	28	4	198	0.0033	19
cJun	20	90	50	48	15.6	0.0008	9

^*a*^Based on the degradation rates of full-length 18-mer RNA substrate in 20 m*M* Tris-HCl, 80 m*M* KCl, pH 7.2, 37°C.

### Mass spectral analysis of RNA18 - GCN4 LZ35 complex formation

**Figure 5:**
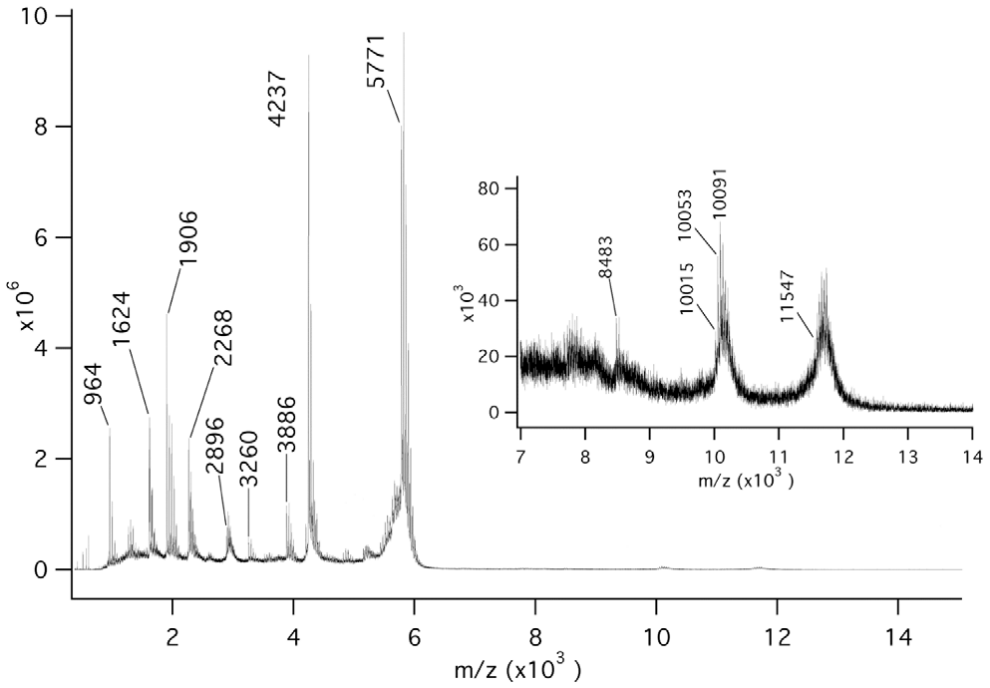
MALDI-MS analysis of LZ35–RNA18 complex formation. The 1 to 14 kDa portion of the MALDI mass spectrum of a mixture of GCN4 LZ35 (69 µ*M*) and RNA18 (68 µ*M*) recorded in negative ion mode using 6-aza-2-thiothymine/citrate as matrix. The signals obtained are explained in Results.

Matrix-assisted laser desorption/ionization (MALDI) mass spectrometry [Fig. 5] showed signal groups starting at 11′547 and 10′015 Da indicating the presence of double-stranded RNA18 and a 1∶1 molar complex of monomeric 35-residue peptide with single-stranded RNA18, respectively. The deviations from the masses expected (11′540 and 10′008 Da) were below 0.1% and thus well within the range of accuracy of the instrument and the associated mass calibration. In both groups, the spacing of the signals with higher mass were m = 38 (dominant, exchange of K for H) and m = 22 (minor, exchange of Na for H), respectively. The alkali cationization of the phosphate backbone of RNA18 could not be suppressed completely despite the use of citrate in the matrix. The lower mass range of the spectrum showed the signals for the monomers of RNA18 (5′771 Da) and LZ35 (4′237 Da) as well as signals for major cleavage products of RNA18 at 1′906 Da (AAUUAC>), 1′624 Da (GGUCU), 1′616 Da (GAAUU>), and 964 Da (AAU>) (where U>, C>, G>, and A> refer to terminal 2′,3′-cyclic phosphates of U, C, G, and A).

Mass spectral analysis showed also that the leucine zipper peptides emerged unaltered from the catalytic process; as true enzymes, they were not oxidized or otherwise modified.

## Discussion

This project started with the assumption that the synthetic 38-residue *retro*-leucine zipper of GCN4 (GCN4 *r*LZ38) could have ribonuclease activity. First assays showed that *r*LZ38, *r*LZ67 [Fig. 1(I d,e)](tetramers: dimers of disulfide dimers), and *r*LZ35 [Fig. 1(I c)](monomer), cleaved the synthetic octadecaribonucleotide RNA18 [Fig. 1(I g)] [Fig. 2(A,B,D)] whereas the HIV enhancer-binding peptide R42, partly contained in *r*LZ67, did not have ribonuclease activity; after a 24-h incubation with R42, RNA18 eluted unchanged from the HPLC column [Fig. 2(C)]. Expectations that the presence of a sequence-specific nucleic acid-binding component may increase the RNA-cleaving activity of the *retro*-leucine zipper domain of fusion peptide *r*LZ67 did not prove to be true. Unexpectedly, the normal, “forward” sequence of the GCN4 leucine zipper (GCN4 LZ35) and the c-Jun leucine zipper (c-Jun LZ36) [Fig. 1(I b,a)] also cleaved the RNA18 substrate (Fig. 3 and Fig. 4). The ribonuclease activity of both normal and *retro*-leucine zipper of GCN4 could be the result of their close structural similarity [9] coupled with the flexibility of enzyme active sites [38]. As the ribonuclease activity of the GCN4 and c-Jun leucine zippers may be of cell biological relevance, all further studies were performed with the normal leucine zippers.

### Substrate specificity

First it was shown that the nuclease activity of the GCN4 leucine zippers was RNA-specific. Besides the synthetic RNA18 [Fig. 2(A,B,D), Fig. 3(C) and Supplementary Fig. S3] they cleaved yeast RNA extract [Fig. S5(B)) and poly(C/U/A/G) (analyzed by thin-layer chromatography, data not shown). They did not cleave DNA and were also inactive in chymotrypsin and trypsin activity assays. Although potential substrates, the dinucleoside phosphates CpG and UpG were not split by LZ35 as shown by HPLC; most likely, their binding to the leucine zipper was too weak.

### Products of catalysis

To determine the nature of the ribonuclease activity of GCN4 LZ35, the cleavage products of RNA18 were separated by HPLC and then characterized by electrospray ionization mass spectrometry. Fig. 3(C) shows that the nucleolytic activity of LZ35 was unspecific. The observation that in 24-h reactions almost all products were obtained as 3′-terminal 2′,3′-cyclic phosphates and that LZ35 was completely inactive in 6 *M* urea, made contamination by RNase A unlikely. Furthermore, a number of products was formed through cleavage of G-A, G-C, G-G, and G-U 3′,5′-phosphodiester bonds ([Fig. 3(C)], from right to left: AAUU>, GGUCUG>, GUCU>, UCUG>, UACCAG>, UCUGC>, GG>, the terminal G, UCU>, G>) providing additional evidence that the ribonuclease activity of LZ35 was intrinsic because contamination by a G-specific ribonuclease such as RNase T_1_ could be excluded. For comparison, digestion of RNA18 by RNase A gave the expected hydrolysis products [Fig. 3(B)] lacking completely fragments formed through cleavage after G.

The location of cleavage sites in context of the double-stranded RNA18 structure ([Fig. 3(C), inset], predicted using MFOLD software [37]), suggests that LZ35 is more catalytically active towards the unstructured regions of the RNA18 molecule.

### Negative controls

In the absence of LZ35 or RNase A, RNA18 was not cleaved [Fig. 3(A)]. RNA18 was also unaffected in assays with R42 (the synthetic 42-residue artificial HIV enhancer-binding peptide, net charge +14) [Fig. 2(C)], bovine serum albumin [Fig. S3(G,H)], glyceraldehyde-3-phosphate dehydrogenase and glucagon (not shown). The ribonuclease activity of GCN4 LZ35 and complete c-Jun protein was not affected by RNasin [Fig. S3(A,B) and Fig. 4(B,C)], a specific inhibitor of the ribonuclease A enzyme family. However, at very high concentration (30-fold above the recommended values) RNasin impaired the activity of GCN4 LZ35 by 60%, most likely caused by unspecific interactions between the leucine zipper and the hydrophobic inhibitor [39]. Most importantly, the ribonuclease activity of GCN4 LZ35 was affected by stepwise replacement of several amino acid residues of the leucine zipper with alanine, unequivocally attributing the observed activity to the GCN4 leucine zipper and excluding possible contamination. Fig. 1(**II**), lower panels, shows that those residues of GCN4 LZ35 that are also found in ribonuclease active sites (Glu, Ser, Lys, Tyr, His) are aligned on one side of the GCN4 leucine zipper [40] possibly representing a binding site of the RNA18 substrate. Table 1 summarizes the effect of replacement of these residues mainly by alanine. The Glu12Ala analogue was completely inactive compared to the wild-type sequence. The ribonuclease activity of the Glu12Gln, Glu13Ala, Ser16Ala and Ser16Thr analogues was strongly reduced as compared with that of wild-type LZ35. The activity assays of the His20Ala mutant showed a time-dependent decrease of the area of the RNA18 substrate peak without detectable formation of cleavage products, suggesting reversible aggregation of the His20Ala-RNA18 complex(es) under the conditions employed (see Results and [Fig. S4(E,F)]). The same aggregation takes place at high concentrations of wild-type GCN4 LZ35 [Fig. S4(B,C)] and at high concentrations of catalytically active aminobenzimidazole [41].

### LZ-RNA complex formation

Complex formation between GCN4 LZ35 and RNA18 was demonstrated by MALDI mass spectrometry [Fig. 5]. MALDI data of noncovalent complexes, however, have to be interpreted with caution because sample preparation and laser action are generally disruptive; in addition, nonspecific clusters of sample constituents can be produced in a dense MALDI plume. Disruption of the LZ35 - RNA18 complex by the MALDI process may have been responsible for the strong signals at 4′237 and 5′771 Da (monomeric 35-residue peptide and single-stranded RNA18, respectively). The intense signal groups starting at 10′015 and 11′547 Da [Fig. 5, inset] indicated the presence of specific noncovalent complexes (monomeric 35-residue peptide - single-stranded RNA18 and double-stranded RNA18, respectively) whereas the exponentially decreasing signal intensity around 8′483 Da was typical for the presence of nonspecific clusters.

### Catalytic conformations of the GCN4 and c-Jun leucine zipper peptides

At present the nature of the catalytically active conformation (coiled coil versus monomer) of the native leucine zippers remains unclear. The leucine zipper of GCN4 exhibits an equilibrium dissociation constant (*K*_d_) of 8 n*M* [42] and therefore 99% of the peptide should be in the dimeric coiled coil state in the conditions of the ribonuclease assays. However, the results of MALDI-MS indicate that monomeric GCN4 LZ35 can also form a 1∶1 molar complex with the RNA18 substrate. Similarly, under the conditions employed disulfide-crosslinked *r*LZ38 and *r*LZ67 [Fig. 1(I d,e)] form stable tetramers while non-crosslinked *r*LZ35 [Fig. 1(I c)] is monomeric [43]. Nevertheless, all three *retro*-peptides exhibit comparable ribonuclease activity [Fig. 2]. The leucine zipper of c-Jun has a *K*_d_ of 448 µ*M* [44] and therefore only 18–24% of the peptide shall be in the dimeric state under the conditions employed [Fig. 4] (60–90 µ*M* peptide concentration).

### Catalytic mechanism

Based on the substrate specificity [Fig. 3(C)], the cleavage of RNA by GCN4 LZ and *r*LZ may involve elements of an RNase T_1_-like mechanism. In RNase T_1_, Glu58/His92 were found to act as the active-site base/acid couple with His40 participating in the electrostatic stabilization of the transition state [45]. Later it was shown that RNase T_1_ was still active when both histidines were replaced by aspartate [46]. It is conceivable that two of the four acidic residues which are located within approximately 5.5 Å on the same face of the GCN4 leucine zipper (Glu8, Asp9, Glu12, Glu13) formed an active site, giving rise to the ribonuclease activity of LZ35 and *r*LZ35. It is also of interest that *r*LZ35 was active despite being undoubtedly monomeric in the conditions of the assay [43]. All this shows that at present suggestions for a mechanism of the cleavage of RNA by GCN4 leucine zippers must remain speculative. This applies also to the RNA cleavage by c-Jun LZ36. Whether the partial sequence identity of the GCN4 and c-Jun leucine zippers (KVEEL versus LEEKV) [Fig. 1(I b and a)] hints to a common active site can not be answered and also raises the question if maximum ribonuclease activity is confined strictly to the sequences of the GCN4 and c-Jun leucine zippers. Most likely, the two leucine zippers, although structurally and functionally related, belong to those “enzyme” families in which active site residues are not conserved [38]. In Fig. 1(**II**), the structure of the region of GCN4 LZ35 in which residues were mutated (lower panels) was compared to that of the corresponding region of c-Jun LZ36 (upper panels). It showed that GCN4 and c-Jun have a reversed polarity of charge distribution in these regions of their leucine zippers.

### Kinetics of the ribonuclease reaction of GCN4 LZ35 and c-Jun LZ36

The cleavage of the RNA18 substrate by the leucine zipper peptides was accompanied by a decrease of the area of the RNA18 peak in the HPLC chromatogram which allowed calculation of rate constants and turnover. Considering the conformational dynamics [47] and the relative lack of substrate specificity of these leucine zipper peptides, the resulting numbers were most likely mean values of several primary, kinetically equivalent cleavages (strong arrows in [Fig. 3(C)]). Chromatographic analysis showed also that initially produced fragments were cut further, demonstrated by the formation of internal cleavage products of RNA18 such as ACC> and GAAUU> [Fig. 3(C)]. The complexity of these secondary cleavages, however, did not allow estimation of their individual kinetic constants.

Based on the degradation rate of full-length RNA18 (decrease of the area of the RNA18 peak with time) the lower boundaries of the second-order rate constants and turnover numbers were *k*_2obs_ = 47 *M* ^−1^ min^−1^ and *k*_obs_ = 0.0024 min^−1^ for GCN4 LZ35 and *k*_2obs_ = 19 *M* ^−1^ min^−1^ and *k*_obs_ = 0.0033 min^−1^ for c-Jun LZ36. The turnover numbers are one order of magnitude lower than those of the zinc finger domain of transcription factor ZFY (*k*_cat_ = 0.023 min^−1^) [12] and group II intron ribozymes (*k*_cat_ = 0.03 min^−1^) [17].

It must be noted that the kinetic data of the two leucine zippers for the cleavage of RNA18 were obtained in test-tube conditions. If active *in vivo*, binding of GCN4 and c-Jun to their DNA targets may affect the catalytic rates of their bZIP leucine zippers. In any case, *in vivo* nuclease activity of GCN4 and c-Jun for the fine regulation of transcription would have to be low, otherwise the result would be energetically costly futile cycles of mRNA synthesis and degradation.

### Summary and Outlook

We have shown in test-tube experiments that the leucine zipper domains of c-Jun and GCN4 have intrinsic ribonuclease activity and that this activity is preserved in full-length 40 kD c-Jun, a component of the transcription activation complex AP-1 [Fig. 4(B, C)], and in the artificial sequence context of the *r*LZ67 fusion protein [Fig. 2(D)]. Based on an analysis of the UniProt database (release 15.1, November 2009), c-Jun and GCN4 are the first known examples of specialized transcription factors possessing ribonuclease activity.

However, it may be difficult to demonstrate that the GCN4 and c-Jun leucine zippers catalyze slow RNA degradation *in vivo*. If this were the case, other bZIP- as well as zinc finger-containing transcription factors [12] may show a similar coupling of transcription activation with slow RNA processing/degradation.

Other non-enzyme proteins possessing weak catalytic activities are scarce and mainly comprise DnaK, the Escherichia coli Hsp70 molecular chaperone that catalyzes the isomerization of specific peptide bonds [48], and antibodies that convert O_2_ to H_2_O_2_ [49] and catalyze ozone formation in bacterial killing and inflammation [50]. In contrast, the number of proteins found to be bi- or multifunctional with more or less equally strong activities is growing rapidly, striking examples being fumarate hydratase, a citric acid cycle enzyme which also acts as tumor suppressor [11], and ERK2, an externally regulated (or MAP) kinase which is also a transcriptional repressor of interferon signaling [51].

## Materials and Methods

### Peptide synthesis and purification

All leucine zipper peptides were synthesized by the solid phase method [52], [53] on an Applied Biosystems 433A Peptide Synthesizer using Fmoc (fluorenylmethyloxycarbonyl) chemistry [54] and were purified by reversed-phase HPLC. The purity of the peptides was verified by amino acid analysis and mass spectrometry. The amino acid analysis after acid hydrolysis of synthetic GCN4 LZ35 was representative for the purity of the synthetic peptides used in this study and gave the following amino acid ratios (theoretical numbers are in parentheses): Asp 3.1 (3), Glu 8.0 (8), Ser 1.0 (1), Gly 1.1 (1), Ala 1.0 (1), Val 3.0 (3), Met 0.9 (1), Leu 7.0 (7), Tyr 0.9 (1), His 1.0 (1), Lys 4.9 (5), Arg 3.0 (3). The mass spectra of the synthetic wild-type peptide and the Glu12Ala, Ser16Ala, and His20Ala analogues are shown in Fig. S1 and Fig. S2 of the electronic supplementary material. Cysteine-containing peptides [Fig. 1(I d,e)] were air-oxidized before purification in 20 mM Tris-HCl, pH 7.2, at 5 to 7°C; oxidation was shown to be complete after 6 to 8 hours using Ellman's reagent.

### Ribonuclease assays of leucine zipper peptides of GCN4 and c-Jun

Ribonuclease activities and kinetic data of the leucine zipper-catalyzed RNA degradation were calculated from the time-dependent decrease of the area of the RNA18 peak compared to the RNA18 peak in peptide-free control samples using HPLC.

All reagents, buffers and column eluents employed in the assays were prepared using ribonuclease-free water (commercial RNase/DNase-free water, DEPC-treated water or ultrafiltered water assayed for the lack of RNA-hydrolyzing activity). In all tests, contaminating ribonuclease activity of plastic ware, reagents and buffers was excluded. The stability of the RNA substrate in the reaction buffer for 48–96 hours was imperative for the validity of all ribonuclease assays.

Synthetic wild-type and mutant GCN4 leucine zippers (30 µ*M*, based on the relative molecular mass of the wild-type LZ35 monomer: 4238.5 Da) and RNA18 (50 µ*M*) were incubated in 12.5 µL of 20 m*M* Tris-HCl and 80 m*M* KCl, pH 7.2, at 37°C for 2 to 24 h. Then 100 µL of 20 m*M* ammonium acetate, with 35 µ*M* bromophenol blue as internal reference, was added and 100 µL of the resulting mixture was fractionated on a Nucleosil C-18 300-5 HPLC column (Macherey & Nagel) using a solvent gradient (first solvent: 20 m*M* ammonium acetate, second solvent: 97% acetonitrile). Uncleaved RNA18 and the cleavage products were detected at 254 nm. Digestions in 50 m*M* potassium phosphate and 50 m*M* KCl gave very similar product patterns.

The assays were repeated in the presence of two concentrations of the recombinant RNase A inhibitor RNasin (5 U and 150 U per 12.5 µL assay solution). The GCN4 LZ35 and RNA18 concentrations were 25 µ*M* and 50 µ*M*, respectively. RNase A (9.35 p*M*) was used as control. Incubation time was 13 h.

The peptide and RNA concentrations in the ribonuclease activity assays of synthetic c-Jun LZ36 [Fig. 4(A)] were 60 µ*M* and 170 µ*M*, respectively, in the Tris-HCl buffer; reaction time was 2 to 24 h.

To detect a possible ribonuclease activity of full-length c-Jun, the commercial recombinant protein (8 µ*M*) was submitted to ultrafiltration on a Millipore Ultrafree-0.5 membrane to increase its concentration to 50 µ*M* and to change the buffer to 20 m*M* Tris-HCl, 85 m*M* KCl, pH 7.2. The final c-Jun and RNA18 concentrations in the assays were 20 µ*M* and 90 µ*M*, respectively, in 10 µL of the Tris-HCl buffer. The assays were performed in presence or absence of 1 U of RNasin. After 48 h at 37°C the reaction mixtures were analyzed by gradient elution from an Eclipse XDB-C18 RP-HPLC column (Agilent, 0.46 cm×15 cm; solvent: 20 m*M* ammonium acetate, pH 7.8/acetonitrile; elution was recorded between volume ratios 98∶2 and 85∶15 at 254 nm; gradient volume: 16 mL). The results are shown in Fig. 4(B,C,D).

### Mass spectral analysis of RNA18 - GCN4 LZ35 complex formation and RNA18 cleavage by LZ35

Complex formation was shown by MALDI mass spectrometry using 6-aza-2-thiothymine/citrate as matrix. Spectra were recorded in negative ion, linear mode on a time-of-flight instrument (AXIMA CFR, Shimadzu/Kratos) equipped with a nitrogen laser (λ = 337 nm, 3 ns pulse width). Both RNA18 and LZ35 concentrations were 69 µ*M* in 20 m*M* ammonium acetate, 80 m*M* KCl, pH 6.5. The volume applied was 1 µL.

The cleavage of RNA18 by GCN4 leucine zippers and *retro*-leucine zippers was analyzed by HPLC-electrospray ionization mass spectrometry ([M-H]^−^). The cleavage experiments were performed in 20 m*M* Tris-HCl/80 m*M* KCl at pH 7.2 and 37°C. Samples (5 µL each) were applied on a Nucleosil 100-3 C-18 HD column (Macherey & Nagel) and eluted using a stepwise gradient from 100% 20 m*M* ammonium acetate to 97% acetonitrile. The masses of the separated RNA18 cleavage products were determined on a Bruker ESQUIRE-LC quadrupole ion-trap instrument.

## Supporting Information

Figure S1 Mass spectrum of synthetic 35-residue leucine zipper of GCN4 representing the purity of the peptides used in this study. Experimental and theoretical compound mass of LZ35 were identical (4′237.9 Da). Spectra were recorded on an API III+ instrument (Sciex, Toronto) and compound masses were calculated using the MacSpec software (Sciex).
(0.14 MB TIF)

Figure S2 Mass spectra of synthetic mutants of GCN4 LZ35. Glu12Ala (A), Ser16Ala (B), His20Ala (C). Theoretical compound masses are 4′179.9 Da (Glu12Ala), 4′221.9 Da (Ser16Ala) and 4′171.9 Da (His20Ala).
(0.50 MB TIF)

Figure S3 Effects of RNasin on ribonuclease activity of LZ35, RNase A and RNase T1. Effects of 0.5 U/µL RNasin on the cleavage of 34 µM RNA18 by 50 µM GCN4 LZ35 (A,B), 1 nM RNase A (C,D), 300 nM RNase T1 (E,F), and 150 µM BSA control (G,H). Reactions were performed for 1.5 h (RNase A), 13 h (LZ35), 23 h (RNase T1), and 36 h (BSA) at 37°C in 20 mM Tris-HCl, 85 mM KCl, pH 7.2. Uncleaved RNA18 elutes after approximately 40 min.
(0.55 MB TIF)

Figure S4 Aggregation of RNA18-LZ complexes in the presence of His20Ala mutant and at high concentrations of wild-type LZ-GCN4. (A) Digestion of 34 µM RNA18 by 50 µM wild-type LZ-GCN4. (B) Aggregation of RNA in the presence of high (500 µM) concentrations of LZ-GCN4. (C) Resolubilization of the RNA pellet obtained at high LZ-GCN4 concentration (panel B). (D) RNA18 was not digested by 50 µM Glu12Ala mutant. (E) Aggregation of RNA18 in presence of 50 µM His20Ala mutant. (F) Resolubilization of the RNA pellet obtained in the presence of 50 µM of the His20Ala mutant (panel E). Reactions were run for 13 h at 37°C in 20 mM Tris-HCl, 85 mM KCl, pH 7.2. Resolubilization in (C) and (F) was performed by 10-fold dilution of the sample in 80 mM sodium phosphate, pH 7.4, followed by 2-min incubation at 65°C prior to HPLC fractionation. The difference in substrate retention times between panels A–C (RNA18 eluted after ∼45 min) and D–F (RNA18 eluted after ∼40 min) are caused by shortening the column equilibration time to optimize the LC analysis time experiments D–F.
(0.40 MB TIF)

Figure S5 Chromatographic analysis of the cleavage of baker's yeast RNA by the GCN4 LZ35 peptide. Incubations were in 50 mM sodium acetate, pH 5, at 25°C. After 24 h, the samples were applied on a Nucleosil C-18 300-5 column (Macherey and Nagel) and eluted using a stepwise gradient from 20 mM ammonium acetate to 97% acetonitrile in 60 min. The starting concentration of RNA in (A), (B), and (C) was ∼22 µM (0.5 mg/mL). (A) RNA blank. (B) RNA + GCN4 leucine zipper (30 µM). (C) RNA + BSA (7.35 µM).
(0.55 MB TIF)
