# Supplementary figures and images for: The Leucine Zipper Domains of the Transcription Factors GCN4 and c-Jun Have Ribonuclease Activity
Source: PLoS One. 2010 May 21;5(5):e10765. doi: 10.1371/journal.pone.0010765 (PMC2874015; doi:10.1371/journal.pone.0010765)

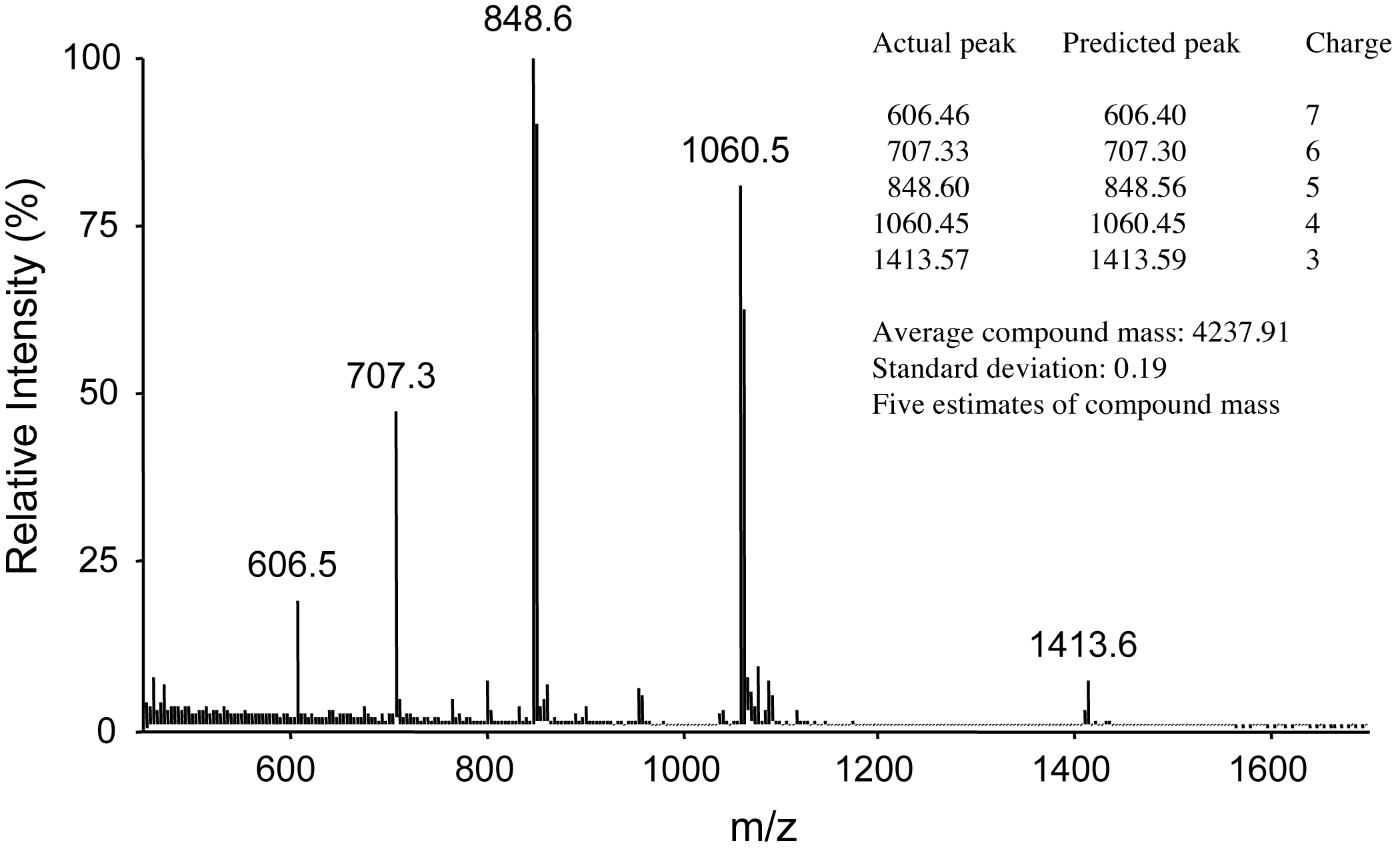

Supplement: Figure S1 — Mass spectrum of synthetic 35-residue leucine zipper of GCN4 representing the purity of the peptides used in this study. Experimental and theoretical compound mass of LZ35 were identical (4′237.9 Da). Spectra were recorded on an API III+ instrument (Sciex, Toronto) and compound masses were calculated using the MacSpec software (Sciex). (0.14 MB TIF) [file pone.0010765.s001.tif]

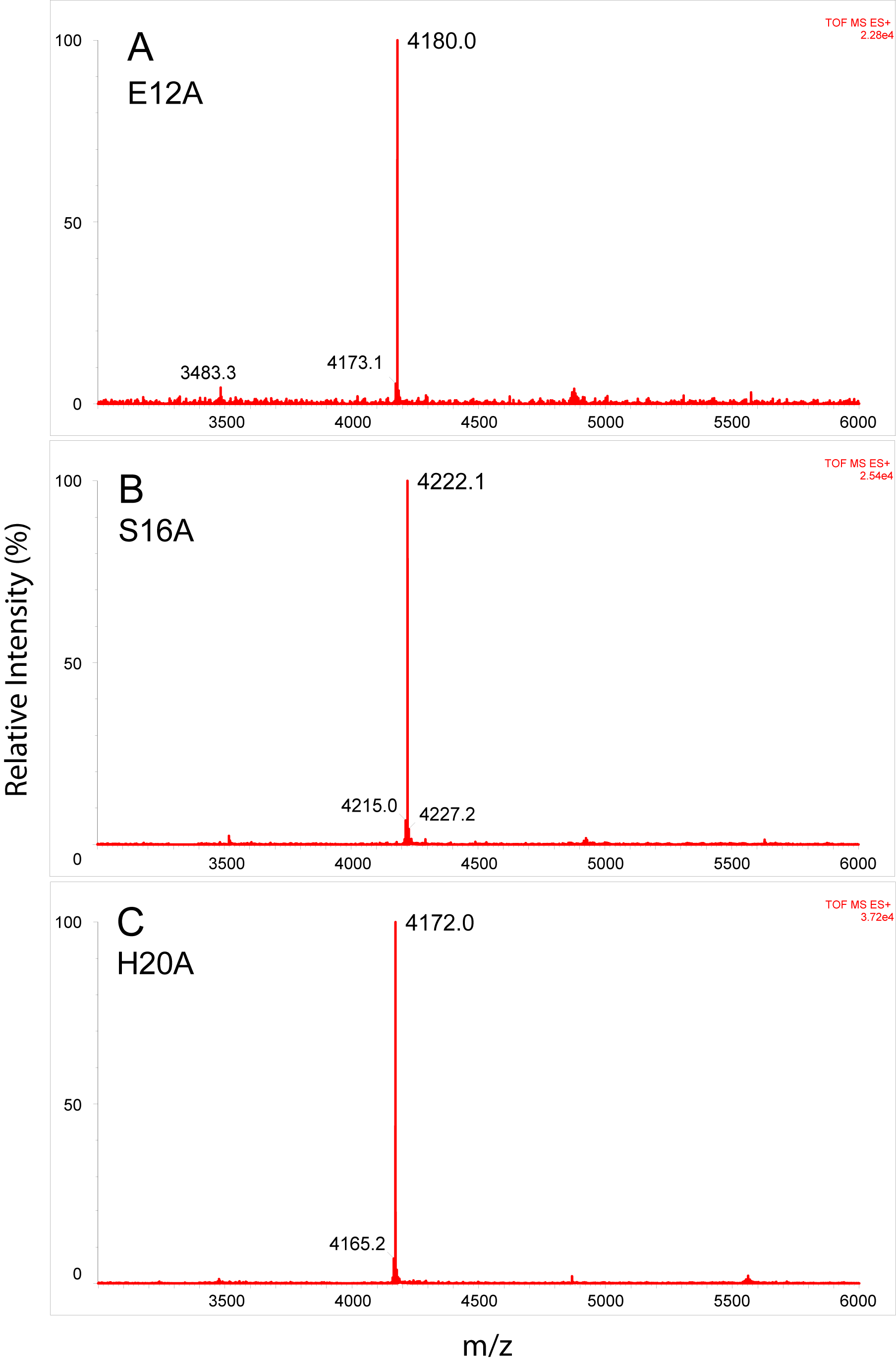

Supplement: Figure S2 — Mass spectra of synthetic mutants of GCN4 LZ35. Glu12Ala (A), Ser16Ala (B), His20Ala (C). Theoretical compound masses are 4′179.9 Da (Glu12Ala), 4′221.9 Da (Ser16Ala) and 4′171.9 Da (His20Ala). (0.50 MB TIF) [file pone.0010765.s002.tif]

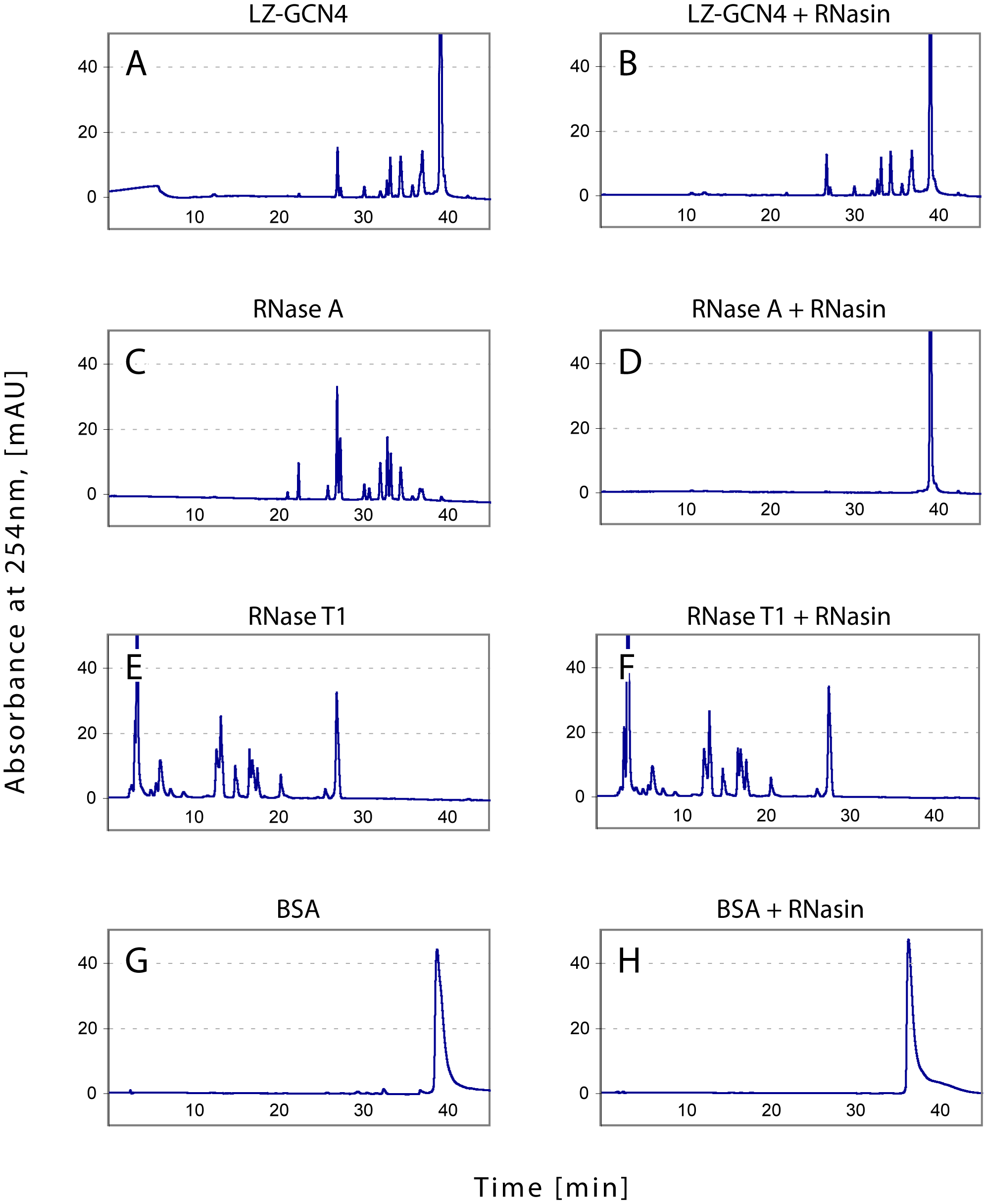

Supplement: Figure S3 — Effects of RNasin on ribonuclease activity of LZ35, RNase A and RNase T1. Effects of 0.5 U/µL RNasin on the cleavage of 34 µM RNA18 by 50 µM GCN4 LZ35 (A,B), 1 nM RNase A (C,D), 300 nM RNase T1 (E,F), and 150 µM BSA control (G,H). Reactions were performed for 1.5 h (RNase A), 13 h (LZ35), 23 h (RNase T1), and 36 h (BSA) at 37°C in 20 mM Tris-HCl, 85 mM KCl, pH 7.2. Uncleaved RNA18 elutes after approximately 40 min. (0.55 MB TIF) [file pone.0010765.s003.tif]

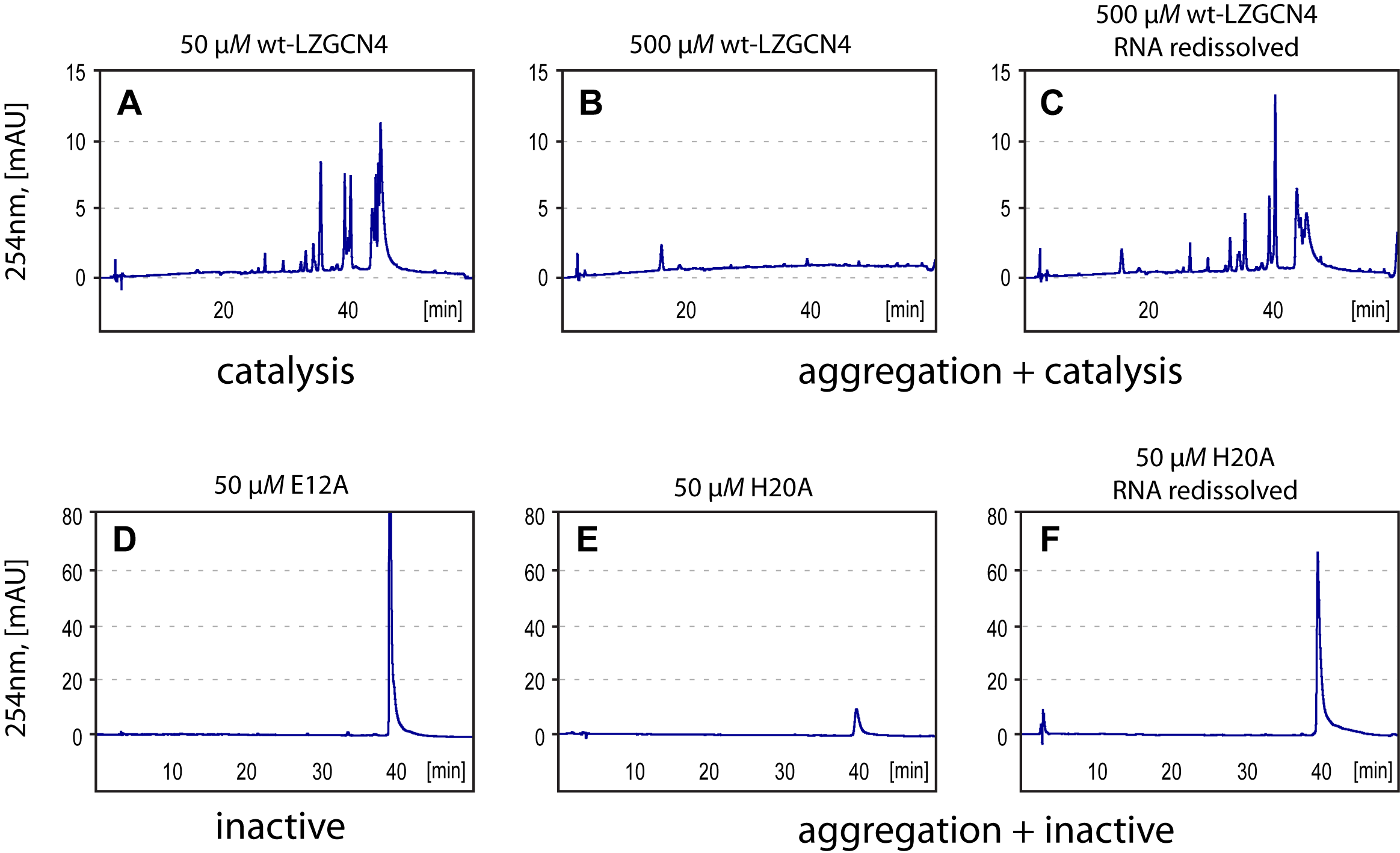

Supplement: Figure S4 — Aggregation of RNA18-LZ complexes in the presence of His20Ala mutant and at high concentrations of wild-type LZ-GCN4. (A) Digestion of 34 µM RNA18 by 50 µM wild-type LZ-GCN4. (B) Aggregation of RNA in the presence of high (500 µM) concentrations of LZ-GCN4. (C) Resolubilization of the RNA pellet obtained at high LZ-GCN4 concentration (panel B). (D) RNA18 was not digested by 50 µM Glu12Ala mutant. (E) Aggregation of RNA18 in presence of 50 µM His20Ala mutant. (F) Resolubilization of the RNA pellet obtained in the presence of 50 µM of the His20Ala mutant (panel E). Reactions were run for 13 h at 37°C in 20 mM Tris-HCl, 85 mM KCl, pH 7.2. Resolubilization in (C) and (F) was performed by 10-fold dilution of the sample in 80 mM sodium phosphate, pH 7.4, followed by 2-min incubation at 65°C prior to HPLC fractionation. The difference in substrate retention times between panels A–C (RNA18 eluted after ∼45 min) and D–F (RNA18 eluted after ∼40 min) are caused by shortening the column equilibration time to optimize the LC analysis time experiments D–F. (0.40 MB TIF) [file pone.0010765.s004.tif]

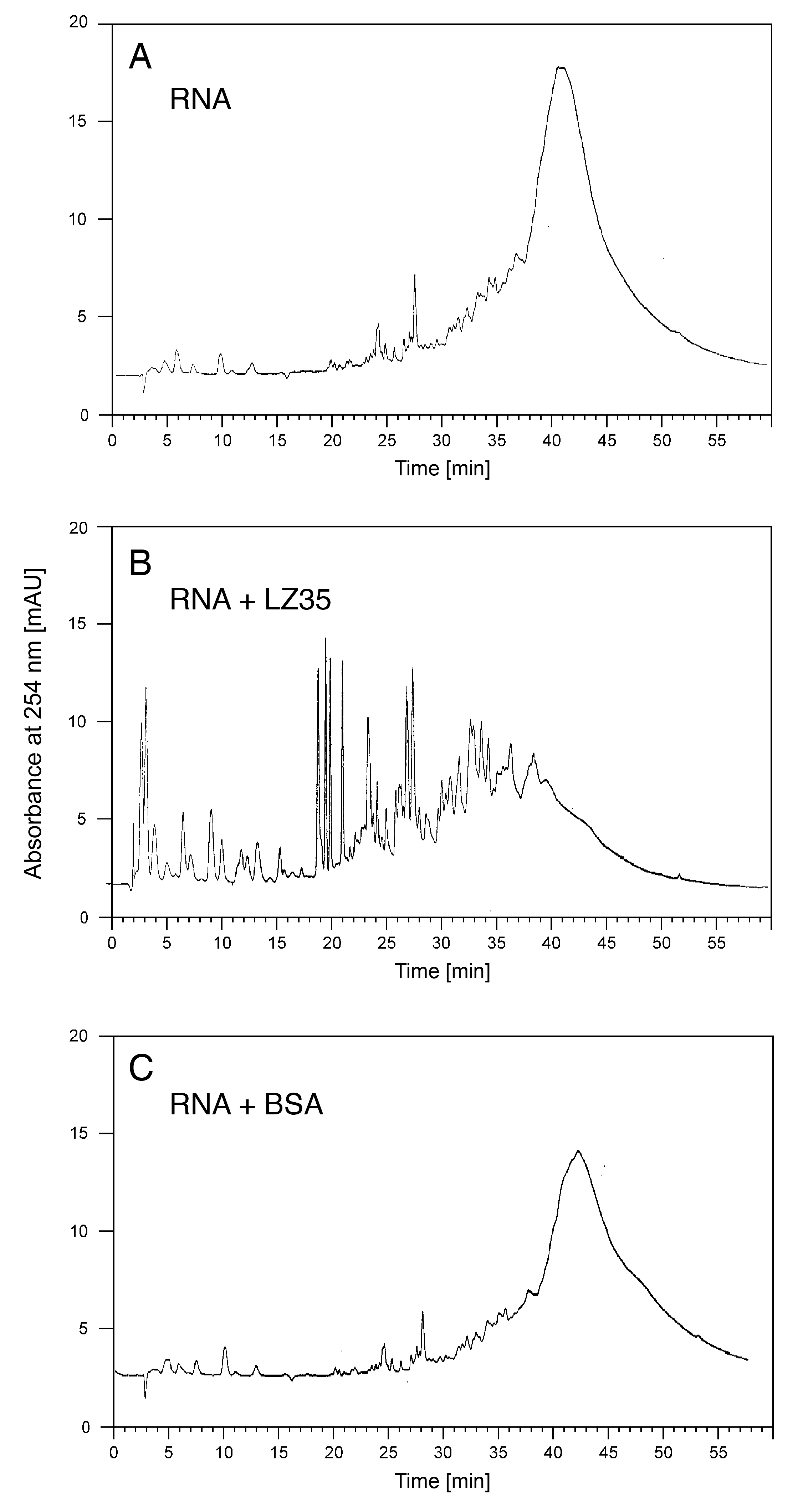

Supplement: Figure S5 — Chromatographic analysis of the cleavage of baker's yeast RNA by the GCN4 LZ35 peptide. Incubations were in 50 mM sodium acetate, pH 5, at 25°C. After 24 h, the samples were applied on a Nucleosil C-18 300-5 column (Macherey and Nagel) and eluted using a stepwise gradient from 20 mM ammonium acetate to 97% acetonitrile in 60 min. The starting concentration of RNA in (A), (B), and (C) was ∼22 µM (0.5 mg/mL). (A) RNA blank. (B) RNA + GCN4 leucine zipper (30 µM). (C) RNA + BSA (7.35 µM). (0.55 MB TIF) [file pone.0010765.s005.tif]
